# Colonization process of *Arabidopsis thaliana* roots by a green fluorescent protein-tagged isolate of *Verticillium dahliae*

**DOI:** 10.1007/s13238-013-0009-9

**Published:** 2014-01-31

**Authors:** Pan Zhao, Yun-Long Zhao, Yun Jin, Tao Zhang, Hui-Shan Guo

**Affiliations:** 1 State Key Laboratory of Plant Genomics and National Center for Plant Gene Research (Beijing), Institute of Microbiology, Chinese Academy of Sciences, Beijing, 100101 China; 2 University of Chinese Academy of Sciences, Beijing, 100049 China

**Dear Editor**,

Verticillium wilt, caused by the soil-borne fungus *Verticillium dahlia* Klep., poses a major threat to a broad host range of more than 400 plant species, including economically important cotton, and causes serious loss of lint yield in China, the USA and may other countries (Bell, 1992; Bejorano-Alcazar et al., 1995; Zhang et al., 2012). The colonization of cotton roots by *V. dahliae* in soil naturally leads to the colonization of vascular tissues in cotton (Garber and Houston, 1966; Gerik and Huisman, 1988). Recently, the development of confocal laser scanning microscopy (CLSM) has significantly increased our knowledge of the colonization process of *V. dahliae* on various plant roots (Eynck et al., 2007; Vallad and Subbarao, 2008; Prieto et al., 2009; Zhang et al., 2012). However, the comprehensive observation of the cotton living root colonization process by *V*. *dahliae* under CLSM is not likely to be practicable due to the large size of cotton roots.

In this study, to better understand the colonization process of V592, which was isolated from cotton in Xinjiang, China, we first inoculated cotton and Arabidopsis with GFP-labeled V592 (V592-GFP1) for an infectivity test in which it was compared with its wild-type isolate, V592. For each isolate, twelve cotton and Arabidopsis seedlings were inoculated with conidia from V592-GFP1 and V592 by unimpaired root dip-inoculation (Supplemental material). Leaf wilt was visually apparent on the cotton leaves at two weeks post-inoculation (wpi), and the whole leaf was dried out, was epinastic and was scorched with stem vascular displaying discoloration (Fig. 1B) by 3 wpi without an obvious difference between the V592-GFP1 and V592 infection (data not shown). Similar disease severity on Arabidopsis caused by V592-GFP1 and V592 was also observed (Fig. 1C). Leaf wilt was observed at 10 days post-inoculation, and the whole leaf was scorched by approximately 3 wpi. These results revealed that the pathogenicity caused by V592-GFP1 did not significantly differ from the wild-type isolate.

**Fig. 1:**
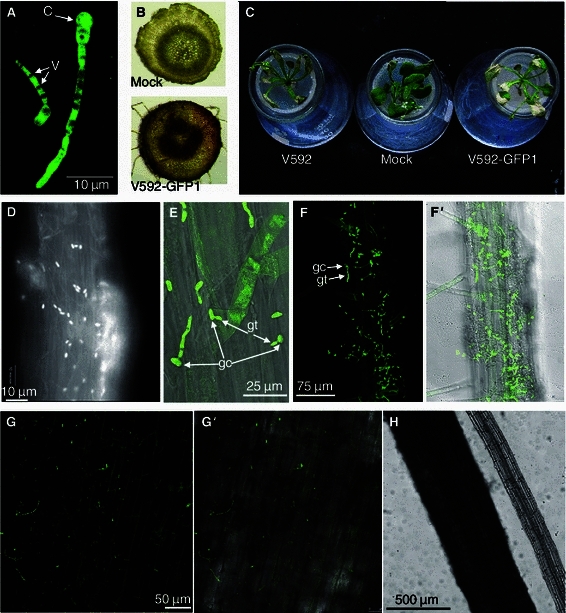
Infectivity of *V* V592-GFP1 and confocal microscopy images of the initial stage root colonization by V592-GFP1 on cotton and Arabidopsis roots. (A) Germinating conidium and hyphae of V592-GFP1. The arrows indicate vacuoles of fungal cells visible as dark areas in the fluorescing cytoplasm. C, conidium, V, vacuole. (B) V592-GFP1-infected cotton stem vascular displayed discoloration compared to mock inoculation. (C) Infectivity of V592-GFP1 relative to V592. Similar leaf wilt symptoms were observed with V592-GFP1 and V592 on Arabidopsis. Photographs were taken two weeks post-inoculation. Mock, mock-inoculation. (D) Conidia-covered Arabidopsis root 6 h post-inoculation (hpi). (E) Germ tubes emerging from one end of the conidium (12 hpi). gc, germinated conidia, gt, germ tubes. (F) Fluorescence image of V592-GFP1 hyphae on a root of Arabidopsis around the root hair zone (24 hpi). (F′) Compound micrograph of bright field transmission and corresponding fluorescence images (same view as F). (G) Fluorescence image of V592-GFP1 hyphae on a root of cotton (24 hpi). Image displays some auto-fluorescence of cotton root epidermal cells. (G′) Compound micrograph of bright field transmission and corresponding fluorescence images (same view as G). (H) Comparison of cotton and Arabidopsis roots under bright field transmission micrograph

Given that its pathogenicity was unaffected, V592-GFP1 was used to monitor the infection processes of *V. dahliae*. CLSM observation started at 6 h after inoculation (hpi). The root surface covered by the conidia at random positions was observed on either the main or lateral roots of both the cotton and Arabidopsis plants (Fig. 1D). By 12 hpi, a small fraction of conidia germinated, with the germ tubes emerging from one end of the conidium (Fig. 1E). The large diameter of the cotton roots limited the optimal confocal laser scanning to each layer of cells during living cotton root observation (Fig. 1G and H). Germination of the germ tubes and growth of the hyphae could only be monitored on the cotton root surface (Fig. 1G). Therefore, the detailed colonization and growth of *V. dahliae* hyphae inside the root at different stages was monitored on Arabidopsis roots, as described below.

By 24 hpi, massive conidia had germinated and extended hyphae on the Arabidopsis root hair zone were observed (Fig. 1F). The growth pattern of most hyphae did not follow the longitudinal grooves of the epidermal cells, and the hyphae wrapping the root surface (Fig. 2A), with only a few germinating hyphae tightly adhering on the root surface either penetrating intercellularly into the epidermal cells (Fig. 2C) and continuing to elongate parallel with the longitudinal axis of the root, or crossing a root epidermal cell before the successful invasion of the junctions of epidermal cells (Fig. 2B). In most cases, the hyphae growth followed a long route along the longitudinal axis before successfully invading the root cortex and further advancing the internal infection (Fig. 2D).

**Fig. 2:**
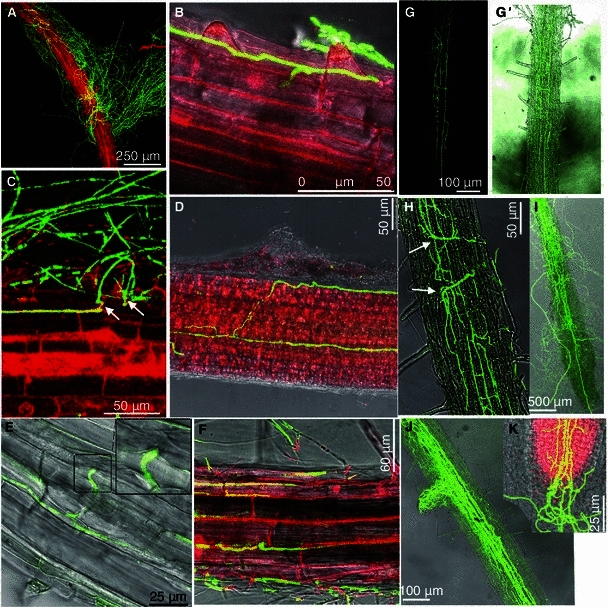
Confocal microscopy images of the advanced stage root colonization by V592-GFP1 on Arabidopsis roots. (A) A mass of hyphae wrapping the root surface and displaying a non-specific growth pattern (2 dpi). (B) Hyphae tightly adhered on the root surface and grew along the longitudinal junction and extended across the transverse to penetrate into epidermal cells intercellularly (2 dpi). (C) The slight swelling of hyphae was followed by formation of a narrow infection peg (arrows) during perforation. (D) Hyphae adhered to the root surface along the longitudinal junction intercellularly across epidermal cells after penetration and internally grew parallel along the longitudinal axis bidirectional (3 dpi). (E) Compound micrograph of bright field transmission and corresponding fluorescence image displaying hyphal swelling intercellularly between the epidermal cell junctions at the site of penetration to an adjacent cell (3 dpi). (F) Hyphal net depicting the cellular structure of the root epidermis displaying intercellular colonization (4 dpi). (G) Hyphal net within the xylem vessel (5 dpi). (G′) Compound micrograph of bright field transmission and corresponding fluorescence images (same view as G). (H) Hyphal swellings (arrows) at the leading edge when hyphae extended transversally (7 dpi). (I) Hyphae extended acropetally up the xylem vessels to the above-ground tissues (10 dpi). Hyphae on the root surface displayed no fluorescence. (J) Hyphae extended to the lateral roots along the vascular tissues (10 dpi). (K) Hyphae extended toward the root tip zone and caused the root cap to collapse (12 dpi). (A–D, F and K) The root was stained with propidium iodide before photographs were taken

The penetration of *V. dahliae* hyphae into the roots was evident by 48 hpi, without the formation of a conspicuous infection structure, such as the appressoria. Through an exhaustive CLSM observation of the penetration site, we observed a slight swelling of the hyphae followed by the formation of a narrow infection peg during perforation (Fig. 2C, arrows). After crossing the transverse junctions of epidermal cells, the hyphae regained their regular size upon reaching the lumen of the epidermal cells (Fig. 2C). The hyphae continued to grow along the longitudinal junction or intercellularly across epidermal cells before growing parallel bidirectionally along the longitudinal axis (Fig. 2D). Intercellular hyphal swelling was observed between the epidermal cell junctions just at the site of the penetration of the elongating hyphae to adjacent cells (Fig. 2E). Whether this hyphal swelling was followed by formation of a penetration peg during this process is not clear. The intracellular colonization of epidermal cells was never observed. Intercellular colonization was evident with the vast majority of the images of the hyphal net depicting the cellular structure of the root epidermis (Fig. 2F).

Upon successful penetration into the epidermal cells, hyphal growth and ramification were sometimes observed within the root cortex in a directed manner toward the central cylinder; however, the extensive internal proliferation of hyphae therein was never observed. Instead, once the hyphae reached the vascular tissue intercellularly through the cortical cells, the hyphae grew and ramified, and a hyphal net within the xylem vessels was observed by 5 dpi (Fig. 2G). The rapid proliferation of hyphae within the xylem vessel cells mainly occurred longitudinally. Hyphal swelling at the leading edge was also visible when the hyphae extended transversally (Fig. 2H).

Although the growth of hyphae was bidirectional within the root, the hyphae quickly progressed acropetally up the xylem vessels to the above-ground tissues (Fig. 2I) and extended to the lateral roots along the vascular tissues by 10 dpi (Fig. 2J). The fungal biomass increased over time exclusively within the xylem vessel cells. For the next two to three days, the hyphae extended toward the root tip zone and caused the root cap collapse (Fig. 2K). However, there was no apparent breakage of the whole root shape. At this stage, the growth of hyphae within the roots still was observable as fluorescent structures, in contrast to the mature hyphae detected on the root surface, most of which did not fluoresce (Fig. 2I). By 2 wpi, the above-ground organs developed typical leaf wilt disease symptoms (Fig. 1C), and a progressive reduction in the detectable hyphae biomass within the root tissue was observed.

The larger size of the cotton roots made it difficult for living cotton root observation using a CLSM. This hampered a comprehensive analysis of each stage of colonization and infection at one particular time point. Vallad and Subbarao (2008) reported the presence of distinct appressoria during the penetration of lettuce roots by *V. dahliae*. However, no appressoria were observed in a recent study of *V. dahliae* on cotton roots (Zhang et al., 2012). Taking advantage of the small diameter of the Arabidopsis root and using exhaustive CLSM observation, we observed slight hyphal swelling and a narrow infection peg during the penetration of the Arabidopsis root cuticle by *V. dahliae* hyphae (Fig. 2C). Hyphal swelling and penetration pegs were observed in a study of *V. longisporum* on oilseed rape plants (Eynck et al., 2007). Within the root, apparent hyphal swelling was usually observed between the epidermal or cortical cell junctions before further penetration (Fig. 2E). Hyphal swelling has been suggested to most likely be due to the accumulation of the cytoplasm in the hyphal tip as a response to the mechanical resistance of the plant tissue (Eynck et al., 2007). Interestingly, only the tight attachment of hyphae to the root surface displayed the possibility of infection, suggesting that substantial interaction between the host and hyphae was necessary for triggering hyphae penetration. Massive hyphae wrapping on the root surface may be necessary as a type of population or “quorum sensing” effect for particular hyphae penetration. The formation of an infection peg suggests an active process of penetration, rather a passive encounter between *V. dahliae* hyphae and a wounded port on the root epidermis. The possible structure and role of the penetration peg in the pathogenicity of *V. dahliae* are worthy of further investigation, which is feasible with the use of T-DNA insertional mutants that were recently constructed by our group (Gao et al., 2010; Zhou et al., 2012).

Although some intercellular hyphal growths and ramifications involving cortical cells after invasion were observed, most hyphal growths were restricted within the cortical tissues. Only a few hyphae could extend in a directed manner toward the xylem vessels where the rapid proliferation of the hyphae took place. These observations, together with the intercellular but not intracellular colonization, were consistent with the studies of *V. dahliae* on oilseed rape, lettuce and cotton roots (Eynck et al., 2007; Vallad and Subbarao, 2008; Zhang et al., 2012), which suggested that plant roots were able to impede the progress of *V. dahliae* hyphae within the cortical tissue either due to intercellular space blockage, active cellular defenses or a combination of both processes. Once the hyphae xylem vessel infection was established, it triggered a typical *Verticillium* resistance in the above-ground plant organs, even though it ultimately caused wilt disease as reported in a recent study (Yadeta et al., 2011). In contrast with *V. dahliae* infection on lettuce roots, where the tips of the lateral roots were the first to invade vascular tissues (Vallad and Subbarao, 2008), we observed that colonies established on Arabidopsis and cotton root hair zones appeared to be the most successful, as they were the first to germinate, display hyphal growth and invade vascular tissues. Nevertheless, despite massive hyphae encompassing the root surface, only a few hyphae successfully invaded the root internal tissues. However, a strategy involving the formidable proliferation of hyphae in xylem vessels compensated for the low hyphae penetration ratio, demonstrating the serious pathogenic threat of *V. dahliae* that led to wilt disease. Unlike in infections with *V. longisporum*, plants maintain their water status (Floerl et al., 2008, 2010) due to a pathogen-triggered plant *VASCULAR-RELATED NAC DOMAIN7* (*VND7*)-dependent de novo xylem formation that involves enhanced water storage capacity and is accompanied by increased plant drought tolerance (Reusche et al., 2012). It has been reported that hop plants tolerant to *V. albo-atrum* infection displayed newly formed xylem cells that were free of tyloses and fungal hyphae (Talboys, 1958). Of the many *Verticillium* species, *V. dahliae* is the only species that blocks vessels and causes cotton wilt under field conditions. This natural plant-fungus pathosystem is interesting and worthy of further investigation focused on the efforts to alleviate the effects of the vascular pathogen on the water status of infected cotton.

## Electronic Supplementary Material

Supplemental Material (PDF 77 KB)
