# Supplementary material for: Colonization process of Arabidopsis thaliana roots by a green fluorescent protein-tagged isolate of Verticillium dahliae
Source: Protein Cell. 2014 Jan 31;5(2):94–8. doi: 10.1007/s13238-013-0009-9 (PMC3956967; doi:10.1007/s13238-013-0009-9)
Supplement: Supplementary file 1 [file 13238_2013_9_MOESM1_ESM.pdf]

Supplemental materials

Materials and Methods

Fungal culture conditions and transformation

A virulent defoliating *V.dahliae* isolate, V592, from cotton originated in Xinjiang, China, was used in this study. This isolate was stored at -80°C in the form of a microconidial suspension in 20% glycerol. Culture was reactivated on potato dextrose agar (PDA) medium (Becton, Dickinson and Company).Conidia germination tests were cultured in liquid Czapek-Dox medium (30 g/L Sucrose, 3 g/L $NaNO_3$, 0.5 g/L $MgSO_4$-$7H_2O$, 0.5 g/L KCl, 100 mg/L $FeSO_4$-$7H_2O$, 1 g/L K_2HPO_4, pH 7.2). *Agrobacterium* transformation was performed as previously described (Gao et al. 2010). Briefly, *A. tumefaciens* strain EHA105, containing a binary vector pSULPH-GFP (Zhou et al. 2009),was grown at 28°C for 2 days in LB Medium (Luria-Bertani Medium) supplemented with kanamycin (50 mg/ml) and was diluted to OD600=0.2 in induction medium (Zhou et al. 2009) in the presence of 200 mM acetosyringone (AS) (BioDee). For co-incubation with a conidial suspension of V592, the culture was transferred to medium containing chlorimuron-ethyl (50 µg/liter) as a selection agent for transformants.

Plant material and liquid culture

Arabidopsis thaliana ecotypes Columbia (Col-0) and upland cotton (cv. Xinluzao NO.16) were used in the infection assays. The Arabidopsis seeds were surface sterilized and stored in wet conditions for 3 days at 4°C for vernalization. The

sterilized seeds were sown on Murashige and Skoog (MS) medium (Duchefa biochemie, the Netherlands) solidified with 0.7% agar (BioWest, Hong Kong). After one week, the seedlings were transferred to a liquid culture vessel filled with 1/4 nutrient solution without organotrophy. These basic nutrients in culture solution consist of 0.02M NH_4NO_3, 0.018M KNO_3, 3mM $CaCl_2$, 1.5mM $MgSO_4$, 1.25mM KH_2PO_4, 0.1mM $FeSO_4$, 0.1mM Na·EDTA, 0.1mM H_3BO_3, 0.1mM $MnSO_4$, 30μm $ZnSO_4$, 5μm KI, 1μm Na_2MoO_4, 0.1μm $CuSO_4$ and 0.1μM $CoCl_2$. Seedlings were grown in a greenhouse at 25°C with16-hlight/8-hdark cycles. The cotton seedlings were planted in MS liquid medium in an environmentally controlled growth room at 26±1°C, 60–70% relative humidity, with a 16-h light/8-h dark cycle.

Root dip-inoculation assay

For the preparation of the inocula, spore suspensions of V592-GFP1 were obtained by passage through 3 layers of cheesecloth (for removing mycelia) after 3 days of culturing in the liquid Czapek-Dox medium and were then adjusted to 5×10^6 conidia/ml with sterile distilled water. For *V. dahliae* inoculations, 10-day-old Arabidopsis plants were incubated in the conidial suspension for 10 min. Control plants were dipped in sterilized distilled water for the same amount of time. The plants were then placed into a nutrient solution. All operations were conducted under sterile conditions. The plants were then placed into a greenhouse under the above mentioned conditions for the observations of the development of symptoms. Cotton root dip-inoculation was performed as described previously (Gao et al. 2010).

Microscopic observations

Microscopic examinations were performed at 6, 12, 24, 48 and 72hours as well as 5 to 10 days post-inoculation. The plant samples were sectioned by hand with a pinhead blade into approximately 2cm lengths and placed on glass slides in drops of water, and then, a cover glass was placed over them. The sections of roots were stained with a 1% propidium iodide (PI) solution in water. Observations were then immediately performed. Microscopic analyses were performed with a Zeiss fluorescence microscope and a Leica TCS SP5 Confocal Laser Scanning Microscope (CLSM). Digital images for GFP were acquired with 488nm excitation and 520nm-540nm emission filters, and confocal settings were optimized for PI with 543nm excitation and 560-580nm emission filters.
